# Characterization of Phosphopantetheinyl Hydrolase from Mycobacterium tuberculosis

**DOI:** 10.1128/Spectrum.00928-21

**Published:** 2021-09-22

**Authors:** Shilpika Pandey, Amrita Singh, Guangli Yang, Felipe B. d’Andrea, Xiuju Jiang, Travis E. Hartman, John W. Mosior, Ronnie Bourland, Ben Gold, Julia Roberts, Annie Geiger, Su Tang, Kyu Rhee, Ouathek Ouerfelli, James C. Sacchettini, Carl F. Nathan, Kristin Burns-Huang

**Affiliations:** a Department of Microbiology and Immunology, Weill Cornell Medicine, New York, New York, USA; b Organic Synthesis Core, Memorial Sloan Kettering Cancer Center, New York, New York, USA; c Department of Medicine, Weill Cornell Medicine, New York, New York, USA; d Department of Biochemistry and Biophysics, Texas A&M University, College Station, Texas, USA; Indian Institute of Science Bangalore

**Keywords:** metallophosphodiesterase, carrier protein, dephosphopantetheinylation, phosphopantetheinyl hydrolase, CoA salvage, *Mycobacterium tuberculosis*

## Abstract

Phosphopantetheinyl hydrolase, PptH (Rv2795c), is a recently discovered enzyme from Mycobacterium tuberculosis that removes 4′-phosphopantetheine (Ppt) from holo-carrier proteins (CPs) and thereby opposes the action of phosphopantetheinyl transferases (PPTases). PptH is the first structurally characterized enzyme of the phosphopantetheinyl hydrolase family. However, conditions for optimal activity of PptH have not been defined, and only one substrate has been identified. Here, we provide biochemical characterization of PptH and demonstrate that the enzyme hydrolyzes Ppt *in vitro* from more than one M. tuberculosis holo-CP as well as holo-CPs from other organisms. PptH provided the only detectable activity in mycobacterial lysates that dephosphopantetheinylated acyl carrier protein M (AcpM), suggesting that PptH is the main Ppt hydrolase in M. tuberculosis. We could not detect a role for PptH in coenzyme A (CoA) salvage, and PptH was not required for virulence of M. tuberculosis during infection of mice. It remains to be determined why mycobacteria conserve a broadly acting phosphohydrolase that removes the Ppt prosthetic group from essential CPs. We speculate that the enzyme is critical for aspects of the life cycle of M. tuberculosis that are not routinely modeled.

**IMPORTANCE** Tuberculosis (TB), caused by Mycobacterium tuberculosis, was the leading cause of death from an infectious disease before COVID, yet the *in vivo* essentiality and function of many of the protein-encoding genes expressed by M. tuberculosis are not known. We biochemically characterize M. tuberculosis’s phosphopantetheinyl hydrolase, PptH, a protein unique to mycobacteria that removes an essential posttranslational modification on proteins involved in synthesis of lipids important for the bacterium’s cell wall and virulence. We demonstrate that the enzyme has broad substrate specificity, but it does not appear to have a role in coenzyme A (CoA) salvage or virulence in a mouse model of TB.

## INTRODUCTION

Ppt, also called P-PantSH, is the nonadenosyl moiety of CoA that is essential for the synthesis of fatty acids, polyketides, and nonribosomal peptides in microorganisms (1). The covalent attachment of Ppt to CPs furnishes them with a free sulfhydryl that is used to link the building blocks for the synthesis of these complex molecules of primary and secondary metabolism. CPs are protein domains of 80 to 100 amino acids that are found in all forms of life either as monomers or as part of large synthases, such as fatty acid synthases (FASs), polyketide synthases (PKSs), and nonribosomal peptide synthetases (NRPSs). CPs from FAS and PKS are known as acyl carrier proteins (ACPs), and those from NRPS are called peptidyl carrier proteins (PCPs). The CPs become functional when posttranslationally modified by phosphopantetheinyl transferases (PPTases), which catalyze the covalent attachment of Ppt to a conserved serine residue of CPs, forming the corresponding holo-CP. Ppt hydrolases remove the Ppt from holo-CP, forming apo-CP. The physiologic function of this activity is not known.

The literature extensively documents PPTases, in comparison to a few reports for Ppt hydrolases (2–8). PPTases are categorized in three families—family I of AcpS-type PPTases, whose substrates include FAS II ACP, family II of surfactin phosphopantetheinyl transferase Sfp-type PPTases, originally found to act on PCP of surfactin synthetase (9) and known to have broad specificity (9–12), and family III of type I integrated PPTases, whose substrates include apo-ACP of type I yeast and fungal FAS megasynthases (13). The known PPTases in Mycobacterium tuberculosis are family I AcpS and family II PptT (13). M. tuberculosis AcpS and PptT have distinct substrates; AcpS substrates include FAS I and FAS II ACPs, whereas PptT substrates include PKS ACP and NRPS PCP. Together, these two PPTases are involved in the production of unusual mycobacterial lipids that function in both cell wall structure and virulence (14). In contrast to AcpS, PptT is essential for the survival of M. tuberculosis *in vitro* and *in vivo* (15).

A Ppt hydrolase activity called ACP hydrolase was first identified in Escherichia coli crude lysates in 1967 (2). Much later, the gene responsible for this activity was identified as *acpH* (3). Purification of overexpressed E. coli AcpH was difficult due to self-aggregation, but the protein was successfully purified under denaturing conditions, and activity on CP substrates *in vitro* was determined (3). AcpH homologs from proteobacteria and cyanobacteria are reported to be active *in vitro* on CPs from FAS, PKS, and NRPS and on peptide substrates (4, 5). The structure of AcpH supports its assignment as a member of the HD (histidine/aspartate) family of phosphodiesterases; mutations in the metal binding residues significantly reduce its enzymatic activity (6). AcpH is not essential in E. coli (3), and its function in the cell remains unclear.

M. tuberculosis contains no orthologs of AcpH, and it was not known how CPs have their phosphopantetheinyl groups removed in M. tuberculosis. In 2019, Ballinger et al. reported that loss-of-function mutations in a conserved hypothetical protein, Rv2795c, conferred resistance to the mycobactericidal actions of a drug-like molecule that partially inhibited PptT (Rv2794c) and that purified, recombinant Rv2795c removed Ppt from the holo-CP AcpM (7). The authors named Rv2795c phosphopantetheinyl hydrolase (PptH). They reasoned that PptH counteracted the action of PptT, rendering the partial inhibitor of PptT mycobactericidal (7).

The crystal structure of PptH was recently solved at 2.5 Å (8), revealing a 4-layer sandwich fold of 12 helices and 10 β-strands, typical of metallophosphoesterases (MPEs). The active site is occupied by a Mn-Fe binuclear center that activates water for nucleophilic attack on the phosphodiester bond and releases Ppt from substrate holo-CPs. The structure is distinct from that predicted for AcpH-type Ppt hydrolases (ATPHs), which are modeled to consist mainly of alpha-helices with few or no β-sheets/strands (8). The lack of structural and sequence similarity between the ATPHs and PptH put PptH into a distinct class, PptH-type Ppt hydrolases (PTPHs).

Here, we have characterized the metallophosphodiesterase activity of M. tuberculosis PptH, identified M. tuberculosis PKS13 as the second known substrate for PptH from M. tuberculosis, and shown that PptH is active on nonnative CPs belonging to FAS (EcACP [E. coli], KpACP [Klebsiella pneumoniae], PaACP [Pseudomonas aeruginosa]) and NRPS (EcEntB [E. coli]) families. Lysates from M. tuberculosis lacking PptH lost the ability to dephosphopantetheinylate holo-AcpM. However, no role could be ascribed to PptH in CoA salvage, nor did the enzyme provide a survival benefit for M. tuberculosis during infection of mice. Current efforts involve investigating the role of PptH in the life cycle of M. tuberculosis that are not routinely modeled, such as transmission.

## RESULTS

### PptH is a metal-dependent phosphodiesterase.

**FIG 1:**
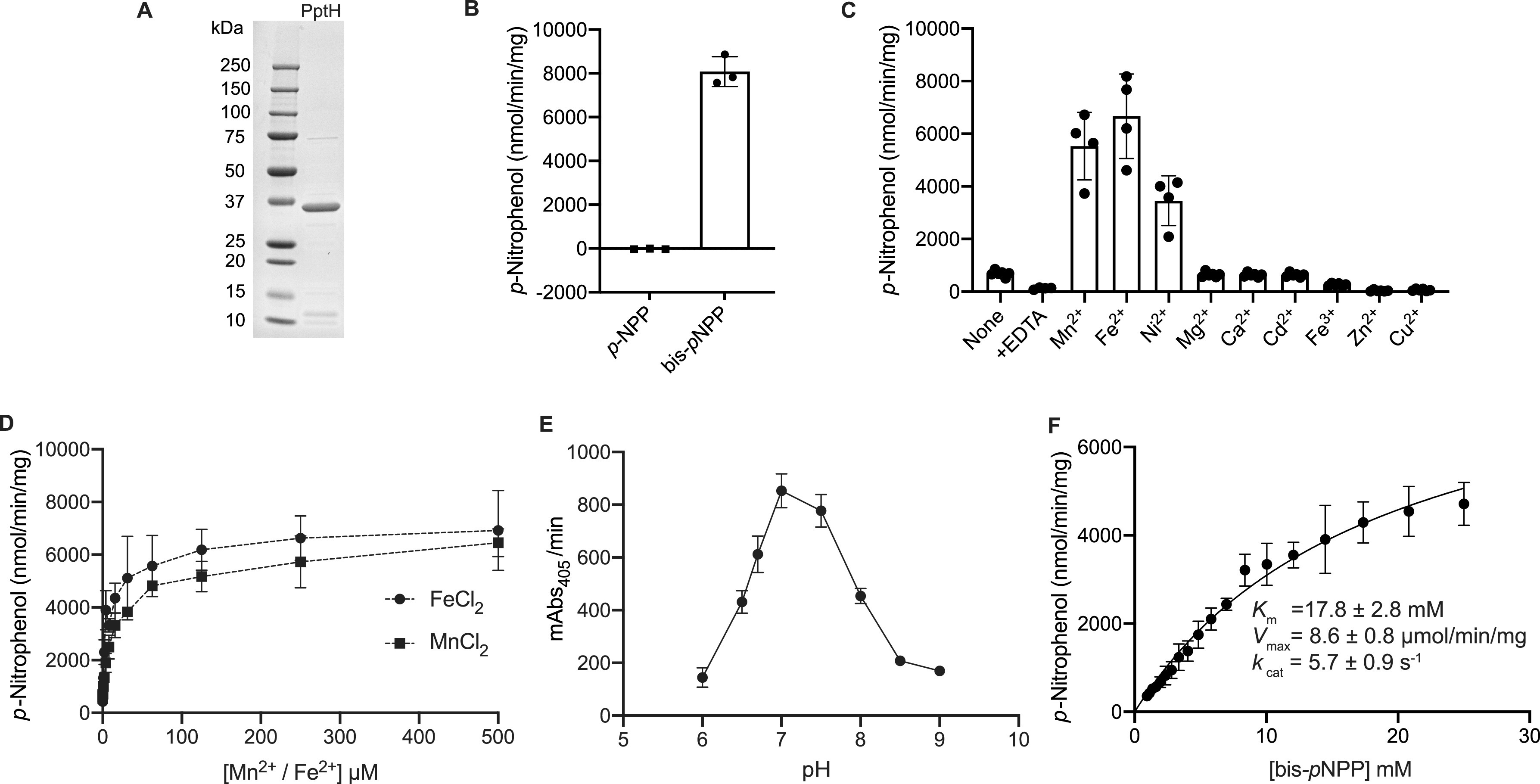
PptH is a metallophosphodiesterase. (A) Purified PptH. Coomassie brilliant blue-stained SDS-PAGE gel of M. tuberculosis PptH (Rv2795c) protein purified from E. coli. (B) PptH is a phosphodiesterase. PptH has phosphodiesterase activity when tested using the phosphomonoesterase and phosphodiesterase substrates para-nitrophenylphosphate (*p*NPP) and bis *p*-nitrophenyl phosphate (bis-*p*NPP), respectively. Reactions were carried out at pH 7.0 with Mn^2+^ and Fe^2+^. (C) Metal dependence of PptH activity. Mn^2+^, Fe^2+^, and Ni^2+^ enhance PptH phosphodiesterase activity. (D) Dependence of PptH phosphodiesterase activity on the concentration of Mn^2+^ or Fe^2+^. (E) Optimal phosphodiesterase activity of PptH at neutral pH. The *y* axis indicates milli-absorbance of the product, *para*-nitrophenol (*p*-NP), over time. (F) The kinetic parameters of PptH for substrate bis-*p*NPP; *K_m_* is 17.8 mM and *V*_max_ is 8.6 μmol/min/mg. Results show means ± standard deviation (SD) from one experiment performed in at least triplicate. All experiments were performed at least two independent times.

PptH (amino acids [aa-] 1 to 310) was overexpressed in E. coli BL21-AI and purified as reported (8) (Fig. 1A). To study the phosphomonoesterase and phosphodiesterase activity of PptH, we screened the artificial substrates *p*-nitrophenyl phosphate (*p*NPP) and bis(*p*-nitrophenyl) phosphate (bis-*p*NPP), respectively. PptH failed to hydrolyze *p*NPP but hydrolyzed bis-*p*NPP to the chromogenic *p*-nitrophenol (*p*NP) product as detected by its absorbance at 405 nm. The reaction was carried out at pH 7.0 in the presence (Fig. 1B) and absence (see Fig. S1A in the supplemental material) of Mn^2+^ and Fe^2+^. Phosphodiesterase activity on bis-*p*NPP was observed in the absence of exogenous metal ions (Fig. 1C; Fig. S1B), probably because of copurification with metal ions (8), as supported by the decrease in activity after dialysis and addition of 2 mM EDTA to chelate residual metal (Fig. 1C; Fig. S1B).

Activity was enhanced in the presence of Mn^2+^ or Fe^2+^ (Fig. 1C; Fig. S1B), both of which were found in the active site of PptH crystals (8), as well as with Ni^2+^ (Fig. 1C; Fig. S1B). As little as 1.0 μM Mn^2+^ or Fe^2+^ enhanced the specific activity of PptH (0.5 μM) by 2-fold or 3-fold, respectively, with maximum activity observed with 62.5 μM Mn^2+^ (8-fold increase) or Fe^2+^ (11-fold increase) (Fig. 1D). Mn^2+^ and Fe^2+^ each enhanced phosphodiesterase activity of PptH to a similar extent.

### Effects of pH and ionic strength.

Hydrolysis of bis-*p*NPP by PptH was tested over the pH range of 6 to 9, using MES (morpholineethanesulfonic acid) as the buffer at pH 6.0 and 6.5 and Tris over the range of 7.0 to 9.0. Phosphodiesterase activity was optimal at pH 7.0 to 7.5 (Fig. 1E) and was enhanced more than 4-fold compared to pH 6 or pH 9. Activity was highest (6 μmol/min/mg) in the absence of added NaCl and decreased with increasing ionic strength (Fig. S1C).

### Kinetic parameters.

Michaelis-Menten kinetic parameters of PptH with bis-*p*NPP were *K_m_* of 17.8 ± 2.8 mM, *V*_max_ of 8.6 ± 0.8 μmol/min/mg, and *k*_cat_ of 5.7 ± 0.9 s^−1^ (Fig. 1F). The high *K*_m_ highlights the low affinity of PptH for the artificial substrate.

### Effect of potential modulators.

Next, we tested the impact on the phosphodiesterase activity of PptH of cyclic GMP (cGMP), ATP, ADP, AMP, cAMP, guanosine tetraphosphate (ppGpp), coenzyme A (CoA) and polyphosphate (polyP). Only polyP and AMP reduced enzyme activity by >50% at 1 mM (97.5% and 66.8%, respectively) (Fig. S2A). The 50% inhibitory concentrations (IC_50_s) of both polyP and AMP were high (109 μM and 456 μM, respectively) (Fig. S2B and S2C), arguing against their potential to serve as physiologic modulators. We cannot rule out the possibility that some of these small molecules serve as substrates for PptH, as is the case for AcpH and Rv0805, another annotated metallophosphoesterase from *M. tuberculosis* (4, 16).

### Probe-based assay for the identification of PptH substrates.

**FIG 2:**
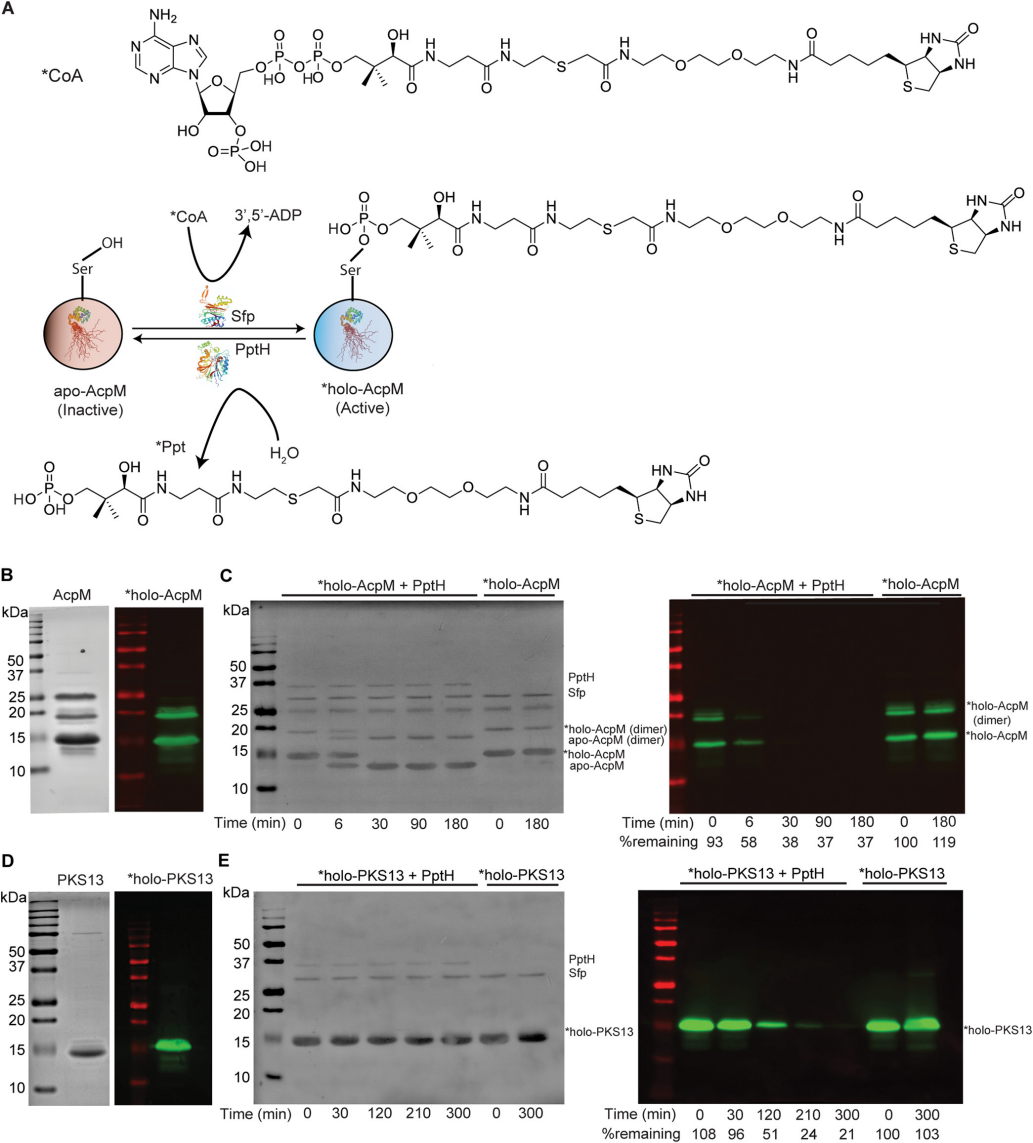
PptH can remove biotin-labeled Ppt from CPs. (A) Biotin-PEG-ACM-CoA probe (*CoA) and proposed reaction scheme used in panels B to E and Fig. 3. (B) Sfp-catalyzed biotin-PEG-ACM-Ppt (*Ppt) labeled holo-AcpM (*holo-AcpM). Ponceau S (left panel) and Western blot using IRDye 800CW streptavidin (right panel). The band at 25 kDa is a contaminant. (C) Time course of PptH-catalyzed hydrolysis of *Ppt from *holo-AcpM. The left panel is Ponceau S staining of membrane, and the right panel is the Western blot of the reaction using IRDye 800CW streptavidin. “%remaining” indicates the percentage of *holo-AcpM (monomer) normalized to no PptH control at *t* = 0 (calculated using ImageJ). (D) Sfp-catalyzed biotin-PEG-ACM-Ppt (*Ppt) labeled holo-PKS13 (*holo-PKS13). Ponceau S (left panel) and Western blot using IRDye 800CW streptavidin (right panel). (E) Time course of PptH-catalyzed hydrolysis of *Ppt from *holo-PKS13. The left panel is Ponceau S staining of membrane, and the right panel is the Western blot of the reaction using IRDye 800CW streptavidin. “%remaining” indicates the percentage of *holo-PKS13 normalized to no PptH control at *t* = 0 (calculated using ImageJ). The experiments were performed three independent times; one representative is shown.

After optimizing PptH phosphodiesterase activity on the nonphysiological substrate bis-*p*NPP, we investigated the activity of PptH on native substrates. The ability of PptH to catalyze the removal of the Ppt moiety from holo-AcpM was shown by SDS-PAGE gel shift of holo- to apo-AcpM and by mass spectrometric analysis of released Ppt (7). Unlike AcpM, most CPs do not show a gel shift upon conversion from holo-CP to apo-CP. To facilitate visualization of this reaction on additional substrates, we synthesized a modified CoA molecule (*CoA) with biotin attached to the sulfhydryl group of Ppt (Fig. 2A). When added to apo-CP by a PPTase, this biotinylated Ppt (*Ppt) served as a probe for Ppt hydrolase activity using a gel-based assay with streptavidin detection (Fig. 2A). P. aeruginosa AcpH was shown to remove coumarin- or rhodamine-modified Ppt from different CPs (17). Our biotinylated probe is reminiscent of maleimide (MAL)-linked biotin-CoA conjugated probes (11, 18), but we replaced the MAL linker with an acetamide (ACM) linker. The biotin-polyethylene glycol (PEG)-ACM-CoA probe (*CoA) avoided a problem encountered with the probe with a MAL linker, where reaction with CPs without addition of PPTase occurred upon denaturation of the samples for SDS-PAGE (not shown).

Upon incubation of the *CoA probe with apo-AcpM and Sfp, a broad-acting PPTase from Bacillus subtilis (9–12), we observed labeling of holo-AcpM (Fig. 2B; *holo-AcpM) as indicated by the streptavidin-reactive band. After removal of unreacted probe and upon addition of M. tuberculosis PptH, we observed time-dependent, PptH-dependent release of the label from *holo-AcpM (Fig. 2C). Mass spectrometry corroborated disappearance of label from the substrate. A peak with a retention time of 4.8 min and mass of 773 Da, corresponding to *Ppt (biotin-PEG-ACM-Ppt), formed upon addition of PptH (Fig. S3). Another peak, with a retention time of 2.7 min and mass of 359 Da, corresponding to unlabeled Ppt, also formed. This likely arose from holo-AcpM in the substrate mixture that copurified with apo-AcpM.

### PptH-mediated dephosphopantetheinylation of M. tuberculosis holo-PKS13.

To determine whether PptH has additional substrates in M. tuberculosis, the ACP domain of M. tuberculosis PKS13, a condensase involved in mycolic acid biosynthesis (19), was expressed in E. coli and purified. PKS13 was converted to labeled holo-PKS13 by Sfp (Fig. 2D; *holo-PKS13), small molecules were removed, and PptH was added. PptH catalyzed the time-dependent removal of *Ppt from *holo-PKS13, as observed for *holo-AcpM (Fig. 2E).

### PptH-mediated dephosphopantetheinylation of heterologous holo-CPs.

**FIG 3:**
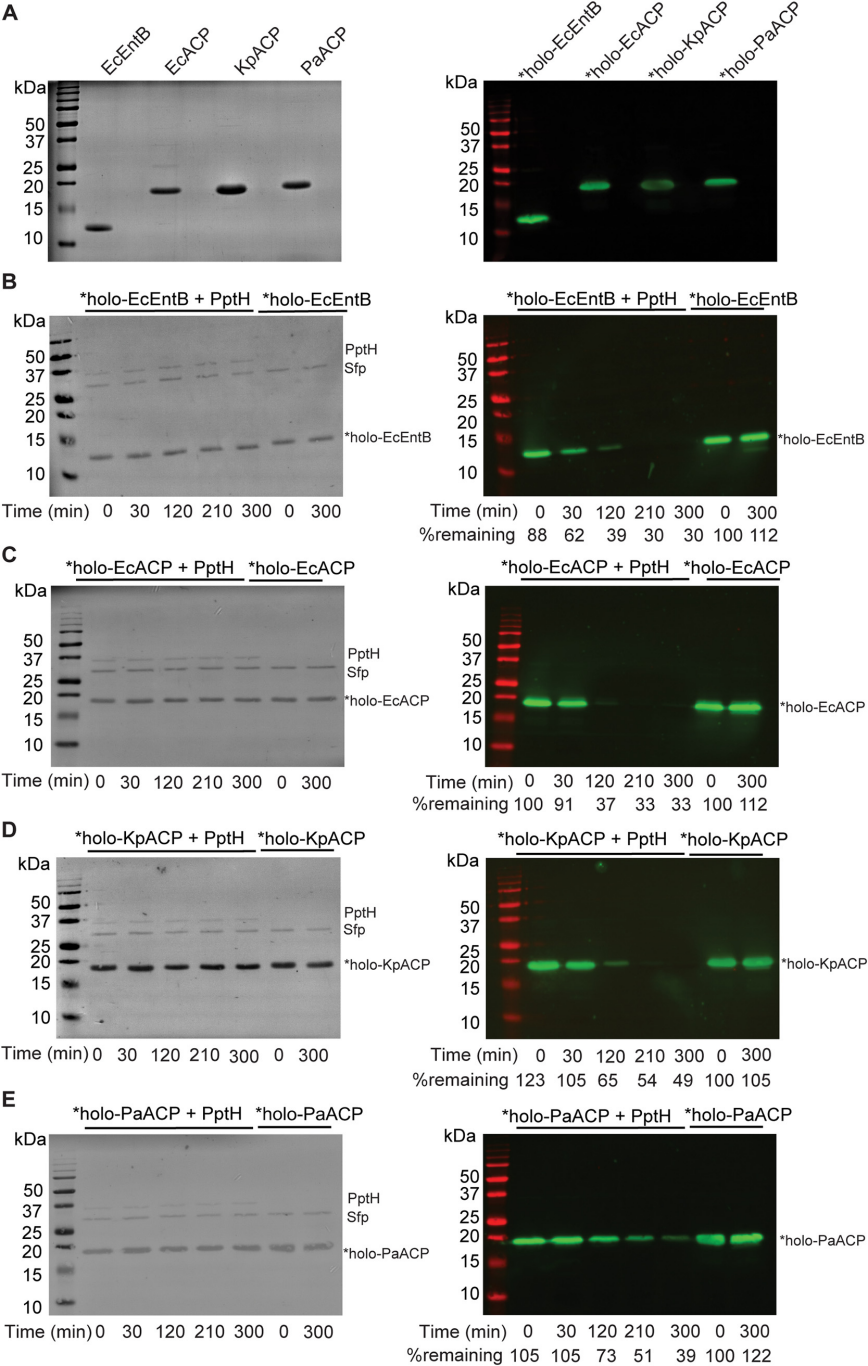
PptH is active on nonnative CPs. (A) CPs from E. coli (EcEntB, EcACP), K. pneumoniae (KpACP), and P. aeruginosa (PaACP) were purified from E. coli, and Sfp catalyzed the transfer of biotin-PEG-ACM-Ppt (*Ppt) to form *holo-CP. SDS-PAGE (left panel) and Western blot using IRDye 800CW streptavidin (right panel). (B to E) Time course of *Ppt-labeled heterologous *holo-CPs from panel A upon addition of PptH. Ponceau S staining (left panel) and Western blot probed with IRDye 800CW streptavidin (right panel). “%remaining” indicates the percentage of *holo-CP normalized to no PptH control at *t* = 0 (calculated using ImageJ). Experiments were performed three independent times; one representative is shown.

AcpHs from E. coli, P. aeruginosa, *Cyanothece*, and Pseudomonas fluorescens show activity on nonnative CPs (3–5). We next asked whether this was true for M. tuberculosis PptH. We cloned, expressed, and purified CP domains from E. coli (EcEntB; EcACP), K. pneumoniae (KpACP), and P. aeruginosa (PaACP). Sfp catalyzed the transfer of *Ppt from *CoA to each of the CPs to form *holo-CPs (Fig. 3A). PptH catalyzed the time-dependent release of *Ppt from each tested *holo-CP (Fig. 3B to E). Thus, PptH is a broad-specificity Ppt hydrolase.

### Nonredundancy of PptH in M. tuberculosis.

**FIG 4:**
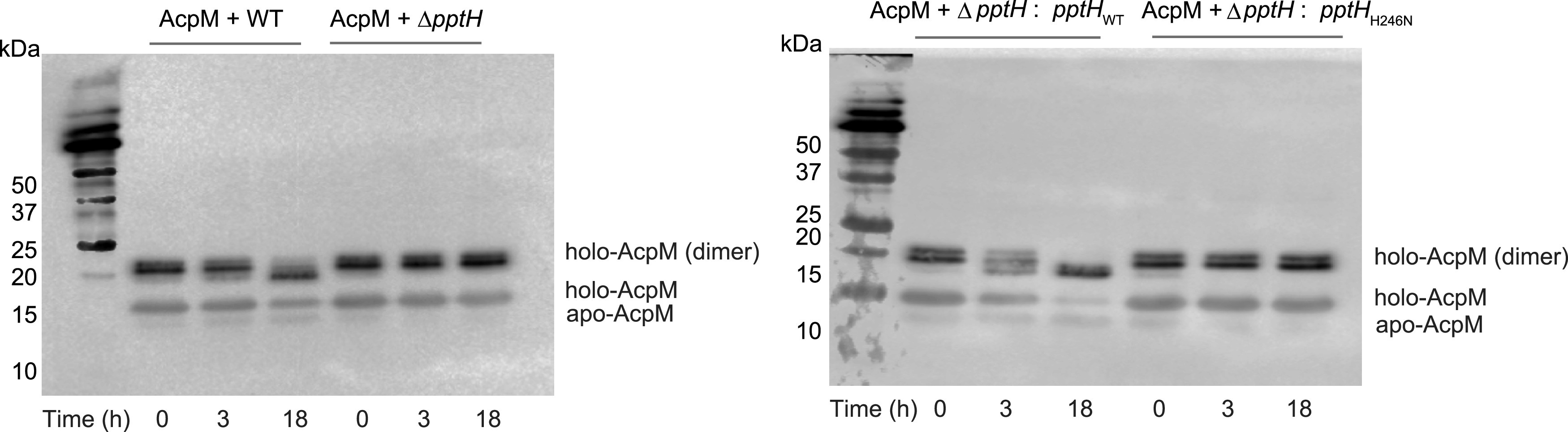
PptH is the only detectable Ppt hydrolase for holo-AcpM in M. tuberculosis lysates. His-tagged holo-AcpM was treated with lysates from WT, Δ*pptH*, Δ*pptH*:*pptH*_WT_, or Δ*pptH*:*pptH*_H246N_ strains of M. tuberculosis, and aliquots were removed over time. His-tagged AcpM gel shift (indicative of Ppt hydrolase activity) was monitored by anti-His immunoblotting. The experiment was performed three independent times; one representative is shown.

AcpH and PptH are structurally distinct Ppt hydrolases that appear to have evolved convergently. AcpH is expressed only in Gram-negative bacteria, while PptH is present in mycobacteria (3, 7, 8). Indirect evidence suggested that AcpH might be the only Ppt hydrolase in E. coli (3). To determine whether M. tuberculosis encodes multiple proteins with Ppt hydrolase activity, we added His-tagged holo-AcpM to whole-cell lysates of M. tuberculosis and asked whether Ppt is removed in lysates from wild-type bacteria and in lysates from M. tuberculosis lacking PptH activity, either because the gene encoding PptH was disrupted or because the cells expressed a mutant of PptH that conferred as much resistance to a PptT inhibitor as did the gene knockout (7). We monitored this reaction by visualizing the gel shift between apo- and holo-AcpM by immunoblot for the His-tag on AcpM. Holo-AcpM shifted to apo-AcpM in whole-cell lysates from wild-type bacteria but was stable in lysates lacking PptH or containing the inactive H246N mutant of PptH (7) (Fig. 4). These results suggest that PptH is the only Ppt hydrolase in M. tuberculosis that can remove Ppt from holo-AcpM under the conditions studied.

### Lack of a role for PptH in CoA salvage.

**FIG 5:**
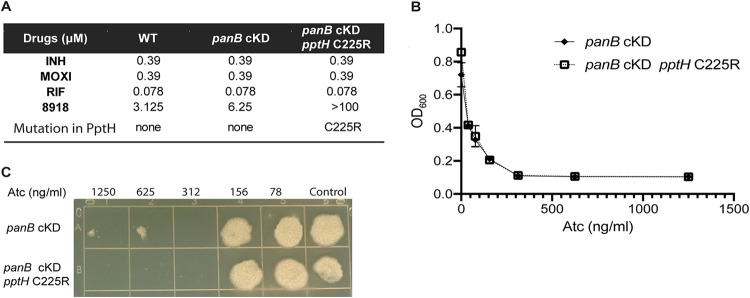
Lack of evidence for a role of PptH in CoA salvage in M. tuberculosis. (A) MIC of M. tuberculosis strains used in the CoA salvage experiment. INH, isoniazid; MOXI, moxifloxacin; RIF, rifampicin. 8918 resistance-conferring mutation C225R in the *panB* cKD strain was confirmed by sequencing of PCR-amplified *pptH* and *pptT* gene products. (B) Atc dose-dependent response of *panB* cKD and *panB* cKD expressing PptH_C225R_ in liquid 7H9 medium (*panB* cKD is TET-OFF; addition of Atc silences transcription). The OD_600_ was measured after day 11 of bacterial growth. Data are means ± SD from one experiment. The experiment was repeated three independent times in triplicate. (C) Atc dose-dependent response of the *panB* cKD strains on 7H10 agar. Cultures of *panB* cKD or *panB* cKD expressing PptH_C225R_ were spotted on 7H10 agar containing Atc. Plates were incubated at 37°C for 2 to 4 weeks. Experiments were performed three independent times; one representative is shown.

Ppt is an intermediate in the biosynthesis of CoA in M. tuberculosis; it is the product of the CoaC-mediated decarboxylation of 4′-phosphopantothenoyl-l-cysteine (20). Ppt is also formed by the PanK-mediated phosphorylation of pantetheine (21). We reasoned that the release of Ppt from CPs by PptH could serve as a salvage pathway to maintain CoA pools in M. tuberculosis. To test this hypothesis, we used a strain of M. tuberculosis in which addition of anhydrotetracycline (Atc) results in conditional knockdown (cKD) of *panB* to deplete the cell’s CoA pool (22). PanB catalyzes the first step in the biosynthesis of CoA by converting 3-methyl-2-oxobutanoate to 2-dehydropantoate, and silencing of *panB* in M. tuberculosis is bacteriostatic (22). We identified *pptH* loss of function mutants in the *panB* cKD strain by selecting for resistant mutants to the reported PptT inhibitor, the amidinourea 8918 (MIC_90_, 3 μM) (7), and resequencing the locus encoding PptT and PptH. We identified an 8918-resistant strain with the point mutation C225R in PptH (Fig. 5A). In the crystal structure of PptH, C225R is located in the active site (8) within hydrogen bond distance to H248, which is involved in metal coordination (8). Mutation from cysteine to arginine is hypothesized to ablate PptH activity due to steric clash, leading to loss of metal binding, similar to what has been observed for other mutants resistant to 8918. We found that the level of PptH C225R is reduced compared to PptH wild type (WT), consistent with the proposed role of C225 in metal binding (Fig. S4). We next monitored the survival of strains upon silencing of *panB* in the background of PptH C225R or in the background of WT PptH, using both liquid and solid agar cultures. Atc-induced transcriptional silencing of *panB* resulted in growth attenuation in both culture formats (Fig. 5B and C), as reported (22). There was no difference in growth between strains with or without functional PptH (Fig. 5B and C). The results suggest that PptH does not play a role in recycling of Ppt in M. tuberculosis under the conditions tested.

### Lack of a phenotype for PptH-deficient M. tuberculosis after aerosol infection of mice.

**FIG 6:**
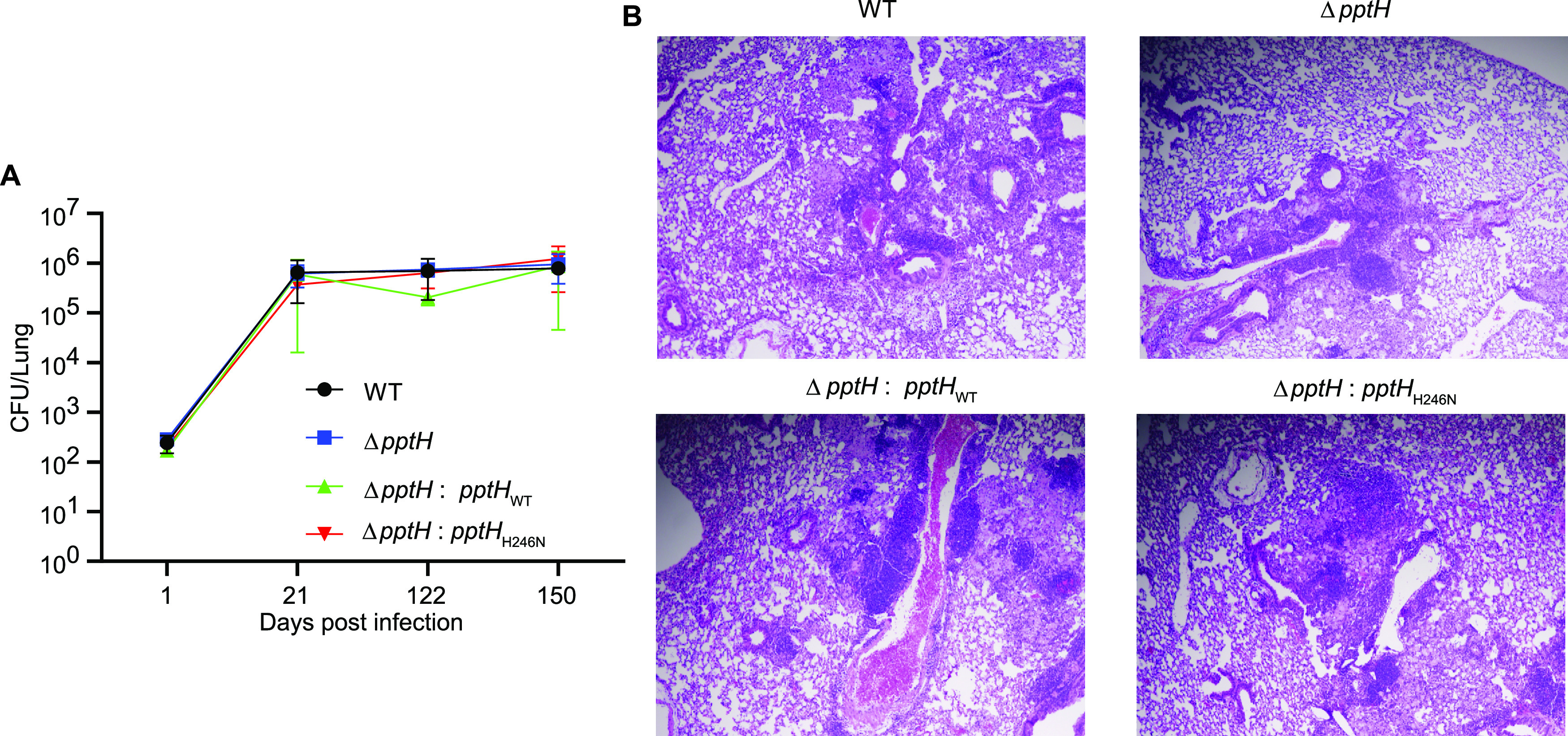
PptH is not required for M. tuberculosis growth and persistence in mice. (A) Colony forming units (CFU) of M. tuberculosis in lungs of C57BL/6 mice. Mice were infected with WT H37Rv, Δ*pptH*, Δ*pptH*:*pptH*_WT_, or Δ*pptH*:*pptH*_H246N_ strains. Data are means ± SD from 5 mice per group per time point (4 mice at day 1) and are representative of two independent experiments. (B) Histopathological examination of M. tuberculosis-infected mouse lungs. Left lobe of mouse lungs infected with WT H37Rv, Δ*pptH*, Δ*pptH*:*pptH*_WT_, or Δ*pptH*:*pptH*_H246N_ strains at day 150 postinfection.

PptH is conserved in mycobacteria, including Mycobacterium leprae with its greatly reduced genome, suggesting that PptH has a critical function that might be manifest in an infected host. To test this, we infected C57BL/6 mice with M. tuberculosis strains WT, Δ*pptH*, Δ*pptH*:*pptH_WT_*, and Δ*pptH*:*pptH*_H246N_. All strains achieved a comparable bacterial burden in lungs through 150 days of infection (Fig. 6A), as well as in spleen and liver (Fig. S5A and S5B). Pulmonary histopathology was also comparable in mice infected with all four strains, as examined at day 150 postinfection (Fig. 6B).

## DISCUSSION

Biologic and biochemical evidence indicated that PptH catalyzes the removal of Ppt from holo-CPs and converts at least one of them into apo-CPs (7). The crystal structure of PptH placed it in a new family of phosphohydrolases and revealed an active site occupied by a Mn-Fe binuclear center (8). In this study, we show that PptH has phosphodiesterase activity dependent on Mn^2+^ and Fe^2+^. E. coli AcpH, which is not homologous to PptH but catalyzes an analogous reaction, is also activated by divalent metal ions (2). The metal ions activate a water molecule to serve as the nucleophile in the hydrolysis reaction. In contrast to the similarity in metal dependence of these two characterized hydrolases, the pH profiles are distinct, with PptH active near neutral pH and AcpH in an alkaline pH range (2). The catalytic efficiency (*k*_cat_/*K_m_*) of PptH is similar to that of PaAcpH (Ppt hydrolase from P. aeruginosa) on the artificial substrate bis-*p*NPP (4). A comparison of catalytic activity of PptH and Rv0805, another M. tuberculosis protein annotated as a metallophosphoesterase with a Mn-Fe binuclear center (8, 16) underscores PptH’s poor affinity for bis-*p*NPP (*K_m_*, 17.8 mM) and its low maximal velocity (*V*_max_, 8.6 μmol/min/mg) compared to those of Rv0805 (*K_m_*, 0.9 mM; *V*_max_, 74 μmol/min/mg). Although PptH phosphodiesterase activity is inhibited by polyP and AMP, this effect is only evident at concentrations above the physiologic range.

To test the activity of PptH on native substrates, we developed a biotin-linked probe (biotin-PEG-ACM-CoA) for an assay to visualize the release of Ppt from CPs. This probe is reminiscent of those developed previously (11, 18) but afforded lower background in our assays. We used the broad-specificity Sfp PPTase to covalently attach the probe to the conserved serine of CPs (18). This assay revealed that M. tuberculosis PptH catalyzes the removal of Ppt from the M. tuberculosis holo-PKS13-ACP domain. PKS13 is essential for mycolic acid biosynthesis and is activated by PptT (14). M. tuberculosis PptH also hydrolyzed Ppt from nonnative holo-CPs, including those from FAS and NRPS, from E. coli, K. pneumoniae, and P. aeruginosa. AcpHs from other organisms are also reported to have broad specificity (3–5).

AcpH appears to be limited to Gram-negative bacteria, and PptH, to mycobacteria (3, 7, 8). M. tuberculosis does not encode a homolog of ATPHs, and since PptH has broad specificity, we reasoned that it may be the only Ppt hydrolase in M. tuberculosis. Whole-cell lysates from wild-type M. tuberculosis hydrolyzed holo-AcpM, whereas lysates from M. tuberculosis lacking PptH or lacking functional PptH (H246N) did not. These results suggest that PptH is likely the only protein responsible for removing Ppt from holo-AcpM in M. tuberculosis and may be the primary Ppt hydrolase in M. tuberculosis. It is possible that under other conditions, including conditions of stress, another hydrolase can compensate for the loss of PptH and perform an analogous function.

The physiological function of Ppt hydrolase activity remains to be determined (3, 7). It is possible that the Ppt released by PptH is incorporated into the CoA biosynthetic pathway, but we saw no evidence that such a route, if it exists, makes a difference to the survival of M. tuberculosis whose growth is limited by reduced synthesis of CoA. Jackowski and Rock found that the rate of turnover of Ppt from holo-ACP is increased when CoA is low (23). However, they also showed that the released Ppt is excreted and not recoverable (24), as has been confirmed (3). It is possible that the Ppt produced by PptH in M. tuberculosis is also excreted and not recoverable. Neither AcpH nor PptH is essential in organisms in which they are encoded (3, 7), and we show here that PptH does not contribute to the survival of M. tuberculosis in a mouse model of infection.

The lack of a phenotype for PptH-deficient M. tuberculosis in a standard mouse model of infection encouraged us to consider what important aspects of the life cycle of M. tuberculosis are not modeled by infection of experimental animals. The major part of the life cycle that goes unstudied in such models is aerosol transmission. Our current efforts to explore the biology of deactivation of holo-CPs by PptH are focused on modeling the changes in state associated with survival of M. tuberculosis in necrotic, hypoxic pulmonary lesions, partial reoxygenation in cavities, aerosolization in fully aerated, rapidly evaporating microdroplets of biologically relevant fluids, and rehydration in pulmonary alveolar lining fluid.

## MATERIALS AND METHODS

### Overexpression and purification of PptH (Rv2795c).

An overnight culture of E. coli BL21-AI expressing truncated PptH (aa 1 to 310) (8) was diluted 1:100 in 2YT (yeast tryptone) broth. The culture was grown to an optical density at 600 nm (OD_600_) of 0.8 followed by cold shock on ice for 20 to 30 min. Inducers (1 mM isopropyl-β-d-thiogalactopyranoside [IPTG] and 0.2% arabinose) were added to the culture, which was kept shaking overnight at 18°C. Bacteria were pelleted by centrifugation and lysed using a French press by resuspending the cell pellet in 50 mM Tris-HCl, pH 8.0, 0.5 M NaCl, 5 mM MgCl_2_, 10% glycerol, and 5 mM imidazole. Cell debris was separated by centrifugation, and the supernatant was incubated with Ni-nitrilotriacetic acid (NTA) beads for 1 h at 4°C. Beads were loaded onto a gravity column and washed with buffers of 50 mM Tris-HCl, pH 8.0, 0.5 M NaCl, 5 mM MgCl_2_, and 10% glycerol containing 10 mM, 20 mM, or 50 mM imidazole. Protein was eluted using 50 mM Tris-HCl, pH 8.0, 0.5 M NaCl, 5 mM MgCl_2_, 10% glycerol, and 250 mM imidazole. The eluted protein was dialyzed in 50 mM Tris-HCl, pH 8.0, 20 mM NaCl, 5 mM MgCl_2_ and 10% glycerol, applied to a HiTrap Q HP anion exchange column, and eluted with a linear gradient of 0 to −500 mM NaCl. The protein yield was ∼2.2 mg/liter of culture. The protein was flash-frozen in liquid N_2_ and stored at −80°C until use.

### Overexpression and purification of carrier proteins and Sfp.

The genes encoding CPs from E. coli (ACP, EntB), K. pneumoniae (ACP), and P. aeruginosa (ACP) were cloned in a pET 28a vector and expressed in BL21-AI (primers are listed in Table S1). M. tuberculosis PKS13 ACP domain was cloned into pMCSG7 (primers are listed in Table S1) and expressed in BL21-AI. Overnight cultures of the transformants were inoculated into fresh 2YT medium and grown to log phase. The culture was put on ice for 20 to 30 min and induced with 0.3 to 1 mM IPTG and 0.2% arabinose for 3 h at 30°C with shaking. AcpM was expressed and purified as reported (25); however, induction was performed at 30°C for 3 h for the labeling experiments with the *CoA probe to increase the proportion of apo-AcpM (26).

Cells were pelleted, resuspended in lysis buffer (50 mM Tris, pH 7.5, 400 mM NaCl, 10% glycerol, 5 mM dithiothreitol [DTT], and 5 mM imidazole) and lysed by French press. Cell debris was separated by centrifugation, and supernatant was incubated with Ni-NTA beads for 1 h at 4°C with shaking. Protein-bound beads were washed with 50 mM Tris pH 7.5, 1 M NaCl, 10% glycerol, 5 mM DTT, and 20 mM imidazole. Protein was eluted in fractions using 50 mM Tris, pH 7.5, 200 mM NaCl, 10% glycerol, 5 mM DTT, and 250 mM imidazole, dialyzed in low-salt buffer (50 mM Tris, pH 7.5, 20 mM NaCl, 10% glycerol, 5 mM DTT) applied to a HiTrap Q HP anion exchange column, and eluted as for PptH. Purification of EcEntB, EcACP, KpACP, and PaACP yielded ∼5 to 10 mg/liter of culture, and PKS13 yielded ∼3 mg/liter of culture. All proteins were flash-frozen in liquid N_2_ and stored at −80°C for further use.

*Sfp* phosphopantetheinyl transferase (*Sfp*) was prepared as described (18). Briefly, a starter culture of BL21-DE3 harboring pET28-Sfp (gift from the Townsend lab) was inoculated into freshly prepared Terrific Broth supplemented with 25 μg/ml kanamycin and grown to an OD_600_ of ∼0.6 at 37°C. Cultures were then cooled in an ice bath for 1 h before overnight induction with 0.5 mM IPTG at 18°C with shaking at 200 rpm. Cell pellets were harvested by centrifugation (4,000 × *g*, 15 min) and stored at −80°C until purification. Cells were thawed in cold lysis buffer (50 mM Tris, 300 mM NaCl, 10% glycerol, pH 8.0) and lysed by sonication. The cell lysate was cleared of debris by centrifugation (27,000 × *g*, 30 min) before incubation with 50% TALON cobalt affinity-resin (4 ml/liter culture) for 1 to 2 h at 4°C in a batch-binding format. The protein-immobilized resin was loaded on a gravity column and washed with lysis buffer containing 10 mM imidazole at room temperature. Bound Sfp was eluted with lysis buffer supplemented with 100 mM imidazole. Fractions containing purified Sfp, as determined by SDS-PAGE with Coomassie staining, were combined and concentrated using an Amicon Ultra 0.5 ml 10K MWCO filtration device (4,000 × *g*, 10 min, 4°C) before dialysis into 50 mM Tris and 5% glycerol, pH 7.5. Protein concentration was determined by Bradford assay.

### PptH phosphomonoesterase and phosphodiesterase activities.

PptH activity was tested using 500 nM PptH and 20 mM phosphomonoesterase substrate *p-*nitrophenyl phosphate (*p*NPP) or phosphodiesterase substrate (bis (*p*-nitrophenyl) phosphate [bis-*p*NPP]) in 50 mM Tris-HCl, pH 7.0, with or without 500 μM MnCl_2_ and 500 μM FeCl_2_ at 37°C in a 384-well plate, measuring absorbance of the *p*-nitrophenol (*p*NP) product at 405 nm for 5 min. The molar extinction coefficient of *p*NP 18,000 M^−1 ^cm^−1^ was used to calculate specific activity.

### Effect of metals on PptH activity.

PptH was purified as described, excluding Mg^2+^ in all steps of the purification, and incubated with 2 mM EDTA for 3 h at 25°C, followed by dialysis against 50 mM Tris-HCl, pH 7.5, 100 mM NaCl, and 10% glycerol before assay with bis-*p*NPP as described above, adding metal ions at a final concentration of 0 to 500 μM or at 500 μM if not otherwise indicated.

### Effect of pH on PptH activity.

PptH activity was tested at 37°C using MES (pH 6 to 6.7) and Tris-HCl (pH 7.0 to 9.0) buffers with 500 nM PptH, 500 μM MnCl_2_, and 20 mM bis-*p*NPP in 50 mM buffers adjusted to the indicated pHs in a 25-μl reaction volume in 384-well assay plates. Reactions were stopped with 40 mM EDTA and 1/10th reaction volume of 2 N NaOH after 3 min. NaOH was added to alkalinize the reaction mixture and convert *p*-nitrophenol to *p*-nitrophenolate, whose absorbance was monitored at 405 nm.

### Effect of ionic strength on PptH activity.

PptH (500 nM) and 20 mM bis-*p*NPP were incubated in 50 mM Tris-HCl, pH 7.0, and 500 μM MnCl_2_ with NaCl (100 to 500 mM), and formation of *p*NP was monitored at 405 nm for 5 min.

### Kinetic parameters with substrate bis-*p*NPP.

Under optimized reaction conditions (50 mM Tris-HCl, pH 7.0, 100 mM NaCl, 500 μM MnCl_2_), 500 nM PptH was added along with increasing concentrations of bis-*p*NPP in 25-μl reaction volumes at 37°C, monitoring absorbance of *p*NP at 405 nm for 5 min. *K_m_* and *V*_max_ were determined by fitting data to the Michaelis-Menten equation with nonlinear regression analysis using GraphPad Prism software.

### Effect of modulators on PptH activity.

The assay with bis-*p*NPP described above included 1 mM ATP, ADP, AMP, polyphosphate (PolyP), ppGpp, 3′,5′-cAMP, 3′,5′-cGMP, or CoA with 500 nM PptH in 50 mM Tris-HCl, pH 7.0, 100 mM NaCl, 500 μM MnCl_2_. The IC_50_ was calculated using GraphPad Prism software.

### General procedure for coupling coenzyme A with iodoacetyl-PEG2-biotin (Fig. S6).

All the solvents were deoxygenated with argon prior to additions. A solution of iodoacetyl-PEG2-biotin (10 mg, 0.0184 mmol, 1 eq.) in DMSO (1 ml) was added to the mixture of CoA tri-lithium salt (15 mg, 0.019 mmol, 1.03 eq) in 8 mM phosphate buffer (pH 7.2, 1 ml). The reaction mixture was stirred in the dark for 6 h at ambient temperature. Reaction progression was monitored by ultra-high-pressure liquid chromatography (UPLC), and the crude mixture was diluted with 3 ml water and was purified directly via preparative high-pressure liquid chromatography (HPLC) on an AutoPure system from Waters Corp. (Milford, MA) using an Atlantis C_18_ column, eluting with a 5 to 40% ACN/10 mM NH_4_HCO_3_ gradient and a flow rate of 20 ml/min for 12-min runs to afford the desired biotin-PEG-ACM-CoA as the ammonium salt (7 mg [33% yield]). Biotin-PEG-ACM-CoA ^1^H NMR spectrum peaks (500 MHz, D_2_O): δ 8.60 (s, 1H), 8.34 (s, 1H), 6.15 (d, J = 6.15 Hz, 1H), 4.80 (m, 2H), 4.51 (m, 2H), 4.32 (m, 1H), 4.18 (brs, 2H), 3.97 (s, 1H), 3.78 (m, 1H), 3.59 to 3.53 (m, 8H), 3.40 to 3.28 (m, 8H), 3.21 (s, 3H), 2.91 (dd, J = 4.7, 12.9 Hz, 1H), 2.70 (d, J = 13.0 Hz, 1H), 2.63 (m, 7H), 2.40 (t, J = 6.5 Hz, 2H), 2.19 (t, J = 7.3 Hz, 2H), 1.64 to 1.49 (m, 4H), 1.31 (m, 2H), 0.86 (s, 3H), 0.73 (s, 3H); ^31^P NMR δ 0.17 to 11.10, −11.20, –11.70, –11.79; electrospray MS calculated for C_39_H_66_N_11_O_21_P_3_S_2_, 1,181.31, found 1,182.72 M+H, 591.83 M/2+H.

### Conversion of CP to biotin-labeled holo-CP.

CPs (each 10 μM) were incubated with 1 μM Sfp in 50 mM Tris-HCl, pH 7.5, 25 mM NaCl, and 15 mM MgCl_2_ containing 10 μM *CoA probe (biotin-PEG-ACM-CoA). The reactions were maintained at 37°C in a heat block for 1 h. Product was visualized using IRDye 800CW streptavidin (LI-COR Biosciences) following SDS-PAGE and transfer to nitrocellulose. Images were scanned using an Azure c600 gel imaging system in the near-infrared region.

### PptH activity on biotin-labeled holo-CP.

Labeled CPs obtained from the Sfp-catalyzed reactions described above were diluted approximately 10-fold with 50 mM Tris-pH 7.5, 100 mM NaCl, 1 mM DTT, and 100 μM MnCl_2_, washed in 10K MWCO Amicon centrifugal filters, rediluted to the original volume, and used as candidate substrates for PptH. The *in vitro* reactions used approximately 10 μM labeled CPs, 500 nM PptH in 50 mM Tris-pH 7.5, 100 mM NaCl, 1 mM DTT, and 100 μM MnCl_2_ at 37°C in a heat block. At 0, 6, 30, 90, and 180 min (AcpM) and 0, 30, 120, 210, and 300 min (PKS13, EcEntB, EcACP, KpACP, PaACP), samples were removed and mixed with Laemmli buffer to stop the reactions. As a control, labeled CPs were incubated without PptH. Samples were run on 15% SDS-PAGE and transferred to nitrocellulose membranes. Following ponceau S staining, the blot was blocked in Pierce protein-free phosphate-buffered saline (PBS) blocking buffer for 1 h at room temperature (RT), rinsed with Tris-buffered saline with Tween 20 (TBST), and probed with IRDye 800CW streptavidin (1:5,000 dilution in Pierce PBS blocking buffer + 0.2% Tween 20) for 45 min at RT. The antibody was removed, the blot was washed 4 times (5 min each time) with TBST, and the image was captured using an Azure c600 gel imaging system in the near-infrared region.

### Mass spectrometry.

Sfp-catalyzed labeled AcpM (biotin-labeled holo-AcpM) was washed with 50 mM Tris-pH 7.5, 1 mM DTT, and 100 μM MnCl_2_ and reacted with 500 nM PptH in 50 mM Tris-pH 7.5, 1 mM DTT, and 100 μM MnCl_2._ At 0, 6, 30, 90, and 180 min, samples were removed and processed for mass spectrometry as follows. Each ultra-high-pressure liquid chromatography (UPLC) sample was prepared by addition of 20 μl of phosphate buffer (pH 7.2) to sample solution (50 μl). Injections were set at 5 μl each on an Acquity UPLC connected to a single quadrupole (SQ) mass detector, C_18_ column (BEH C_18_ column, 1.7 μm, 2.1 × 100 mm) from Waters Corp. (Milford, MA) using the gradient elution system with 2 to 25% acetonitrile in water (both containing 0.01% formic acid) for an 8-min run.

### Test for nonredundancy of PptH.

M. tuberculosis cell lysates were prepared by growing WT, Δ*pptH*, Δ*pptH*:*pptH*_WT_, and Δ*pptH*:*pptH*_H246N_ strains in 7H9 medium (40 ml) to an OD_600_ of 0.8. Cells were pelleted and washed twice with PBS-0.02% tyloxapol. Pellets were resuspended in buffer (50 mM Tris-pH 7.5, 100 mM NaCl, 1 mM DTT, and 1 mM phenylmethylsulfonyl fluoride [PMSF]), and ∼200 μl of zirconium beads was added for bead beating 3 times for 30 s each time at 400 × *g* with 1-min intervals on ice. The lysates were separated from the beads by centrifugation at 800 × *g* for 5 min, and supernatant was passed through a spin-X centrifuge tube filter (0.22-μm cutoff). Reactions (200 μl total volume) used 18 μg protein of lysate from each strain and 1.5 μg holo-AcpM in 50 mM Tris-pH 7.5, 100 mM NaCl, 1 mM DTT, and 100 μM MnCl_2_. After 0, 3, and 18 h of incubation at 37°C, samples were removed and mixed with Laemmli buffer for SDS-PAGE followed by immunoblotting with anti-His antibody (Thermo Fisher Scientific; MA1-135).

### PptH and CoA salvage pathway.

M. tuberculosis *panB* cKD was a kind gift of V. Mizrahi (22). To select for PptH-inactivating mutants in the *panB* cKD background, the *panB* cKD strain was grown in 7H9 medium to the exponential phase and plated on 7H10 agar plates with the amidinourea PptT inhibitor 8918 (1-[(2,6-diethylphenyl)-3-*N*-ethylcarbamimodoyl]urea) at final concentrations of 12 μM (4× MIC) and 25 μM (8× MIC). Plates were incubated for 3 to 4 weeks at 37°C, and the surviving colonies were isolated. Colonies were expanded. We extracted DNA from strains with confirmed resistance to 8918 but without resistance to isoniazid (INH), rifampicin (RIF), or moxifloxacin (MOXI) and sequenced the region of the genome encoding *pptH* and *pptT* (*rv2795c* and *rv2794c*). We selected a clone with the point mutant PptH C225R. To test the effect of transcriptional silencing of Tet-OFF *panB* cKD and *panB* cKD *pptH* C225R, cultures were grown in 7H9 medium with 0 to 1,250 ng/ml of anhydrotetracycline (Atc). At day 11, the OD_600_ was measured. From the same inoculating cultures containing Atc, 10 μl was spotted on 7H10 agar plates and incubated for 2 to 4 weeks. In a separate experiment, we also spotted strains grown in the absence of Atc onto 7H10 agar plates containing 0 to 1,250 ng/ml Atc. These experiments gave the same results.

### Role of PptH in mouse infection.

The WT, Δ*pptH*, Δ*pptH*:*pptH*_WT_, and Δ*pptH*:*pptH*_H246N_ strains were grown as suspension cultures in 7H9 medium containing 0.5% glycerol and 0.05% Tween 80 and were harvested while in the logarithmic growth phase. The bacteria were pelleted by centrifugation; the pellet was resuspended in phosphate-buffered saline (PBS) supplemented with 0.05% Tween 80 and was centrifuged at 800 rpm for 10 min. The supernatant, containing a single cell suspension of M. tuberculosis, was diluted in PBS for infection via the respiratory route in an aerosol infection apparatus in a biologic safety level 3 facility. The apparatus is a self-contained inhalation exposure system (IES) from Glas-Col (model 099C A4212; Terre Haute, IN) that employs a Venturi-nebulizer unit to expose mice to aerosolized M. tuberculosis. The 8-week-old female C57BL/6 mice (Jackson Laboratory) inhaled 100 to ∼200 CFU during a 40-min nebulization. Groups of 5 mice (4 on day 1) were euthanized with CO_2_ at days 1, 21, 122, and 150. At each time point, lungs, liver, and spleen were harvested. Each organ (except the upper lobe of the left lung) was homogenized, serially diluted, and plated for determination of CFU on 7H10 agar containing 10% oleic acid-albumin-dextrose-catalase (OADC) (Difco) and 0.5% glycerol, and the colonies were counted at 3 weeks. For histopathology, at each harvest time point, the upper lobe of the left lung was fixed in 10% formalin in PBS for 24 h at room temperature, followed by embedding, sectioning, staining with hematoxylin and eosin, and photomicroscopy.

### Data availability.

All data are contained within the manuscript.
