# Supplementary material for: Characterization of Phosphopantetheinyl Hydrolase from Mycobacterium tuberculosis
Source: Microbiol Spectr. 2021 Sep 22;9(2):e00928-21. doi: 10.1128/Spectrum.00928-21 (PMC8557913; doi:10.1128/Spectrum.00928-21)
Supplement: SUPPLEMENTAL FILE 1 — Supplemental material. Download SPECTRUM00928-21_Supp_1_seq10.pdf, PDF file, 1.5 MB [file spectrum00928-21_supp_1_seq10.pdf]

Supplementary figure 1

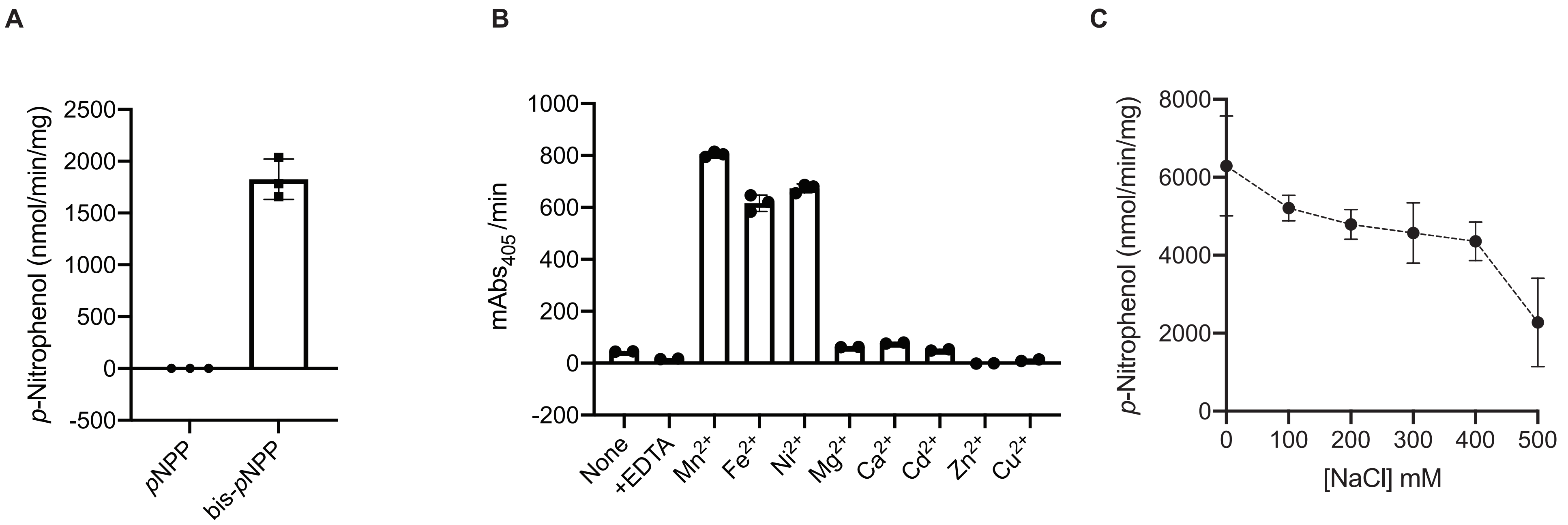

Figure S1. A) PptH catalyzes the hydrolysis of bis-*p*NPP in the absence of Mn^{2+} and Fe^{2+}. B) PptH phosphodiesterase activity with various metal ions at pH 7.5. The y-axis indicates milliAbsorbance of the product, *p*-NP, over time. C) PptH phosphodiesterase activity decreases with increase in ionic strength. Data are means ±SD of one experiment in triplicate (duplicate for None, +EDTA, Mg^{2+}, Ca^{2+}, Cd^{2+}, Zn^{2+}, Cu^{2+}) and performed two independent times.

Supplementary figure 2

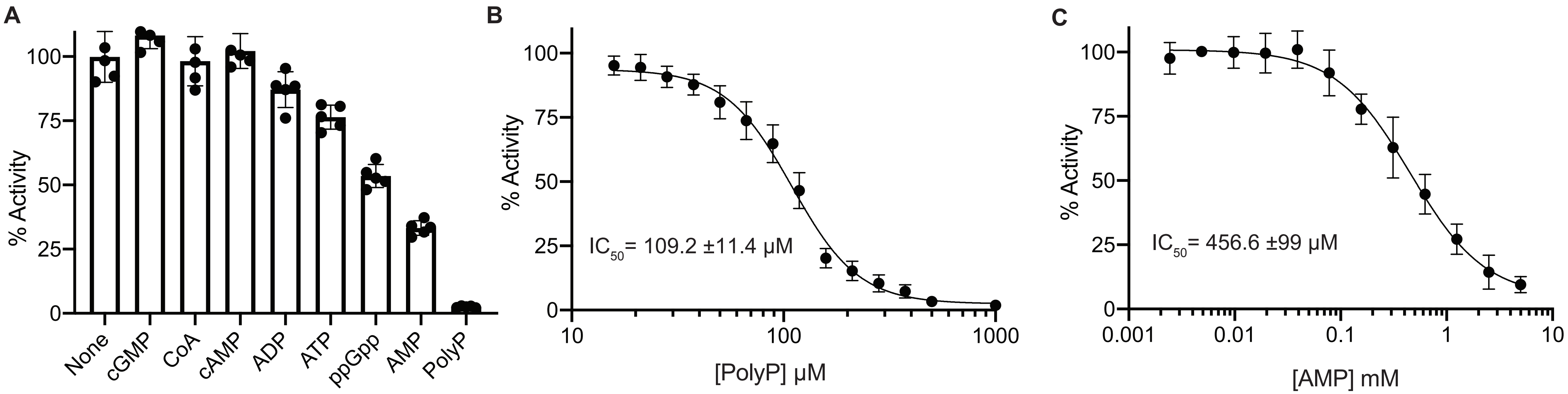

Figure S2. Screening of potential modulators of PptH activity *in vitro*. A) Physiologically relevant molecules were tested for ability to modulate phosphodiesterase activity of PptH in an *in vitro* reaction. Different modulators were added at a final concentration of 1 mM in a reaction with bis-*p*NPP and formation of *p*NP product was monitored at Abs 405 nm. B and C) IC_{50} determination of PptH inhibitors PolyP and AMP. IC_{50} was calculated using non-linear regression curve fit analysis. Data shown are ±SD of one experiment performed in at least triplicate. All the experiments were performed two independent times.

Supplementary figure 3

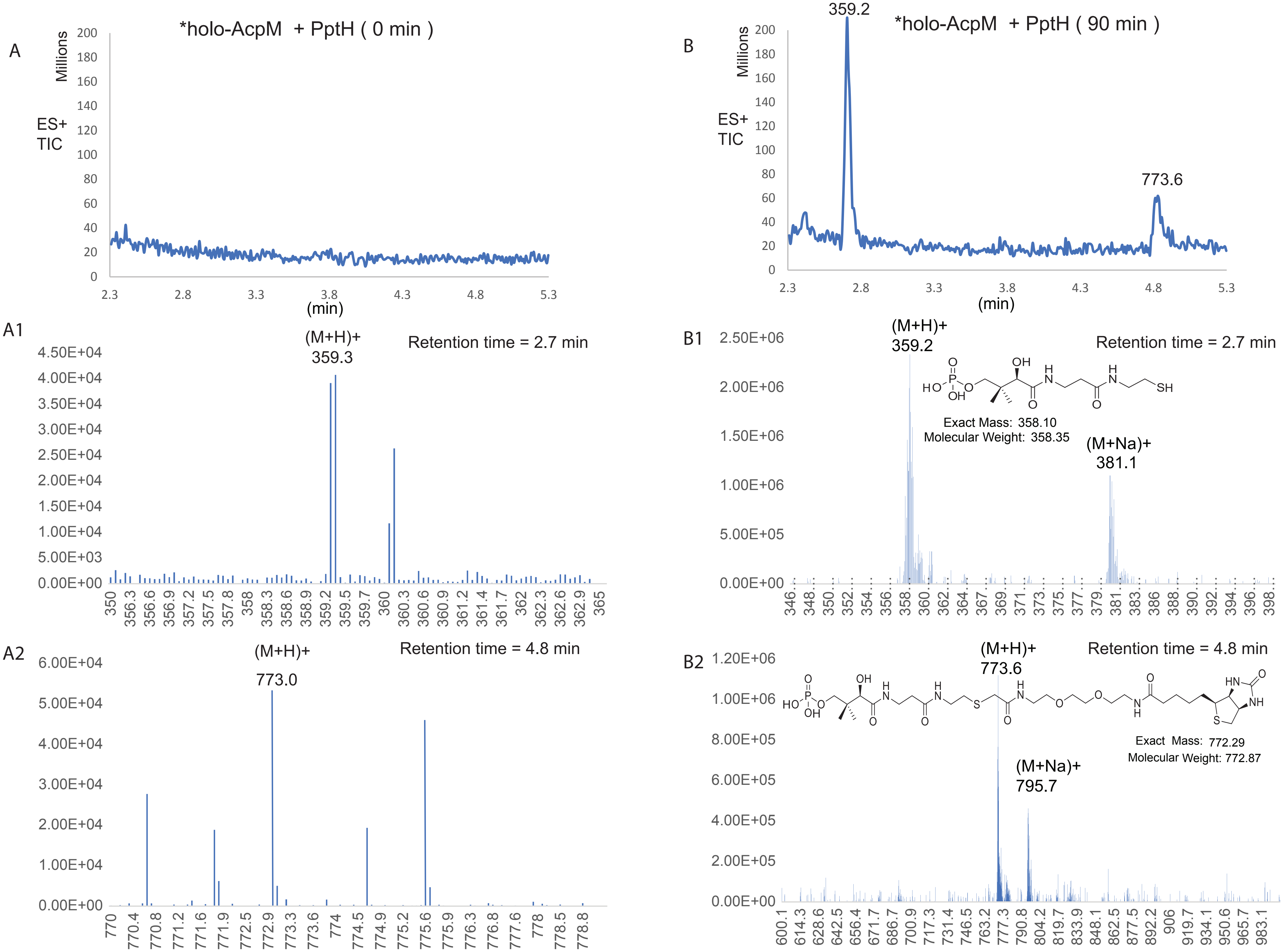

Figure S3. Mass spectrometry of PptH-catalyzed removal of *Ppt from *holo-AcpM. A-D) LCMS chromatograms charting the monitored enzymatic reactions using positive mode mass detection. The reactions were carried out for 0, 6, 30, 90 and 180 min. The lower chromatograms represent retention times of 2.7 and 4.8 min and correspond to the peaks with masses of 359 and 773 respectively. The superimposed structures (3B1, 3B2) depict both products that correspond to those masses. In addition to the $(M+H)^+$ ion, at times the $(M+Na)^+$ ion was detected as well. Experiments were performed two independent times.

Supplementary figure 3

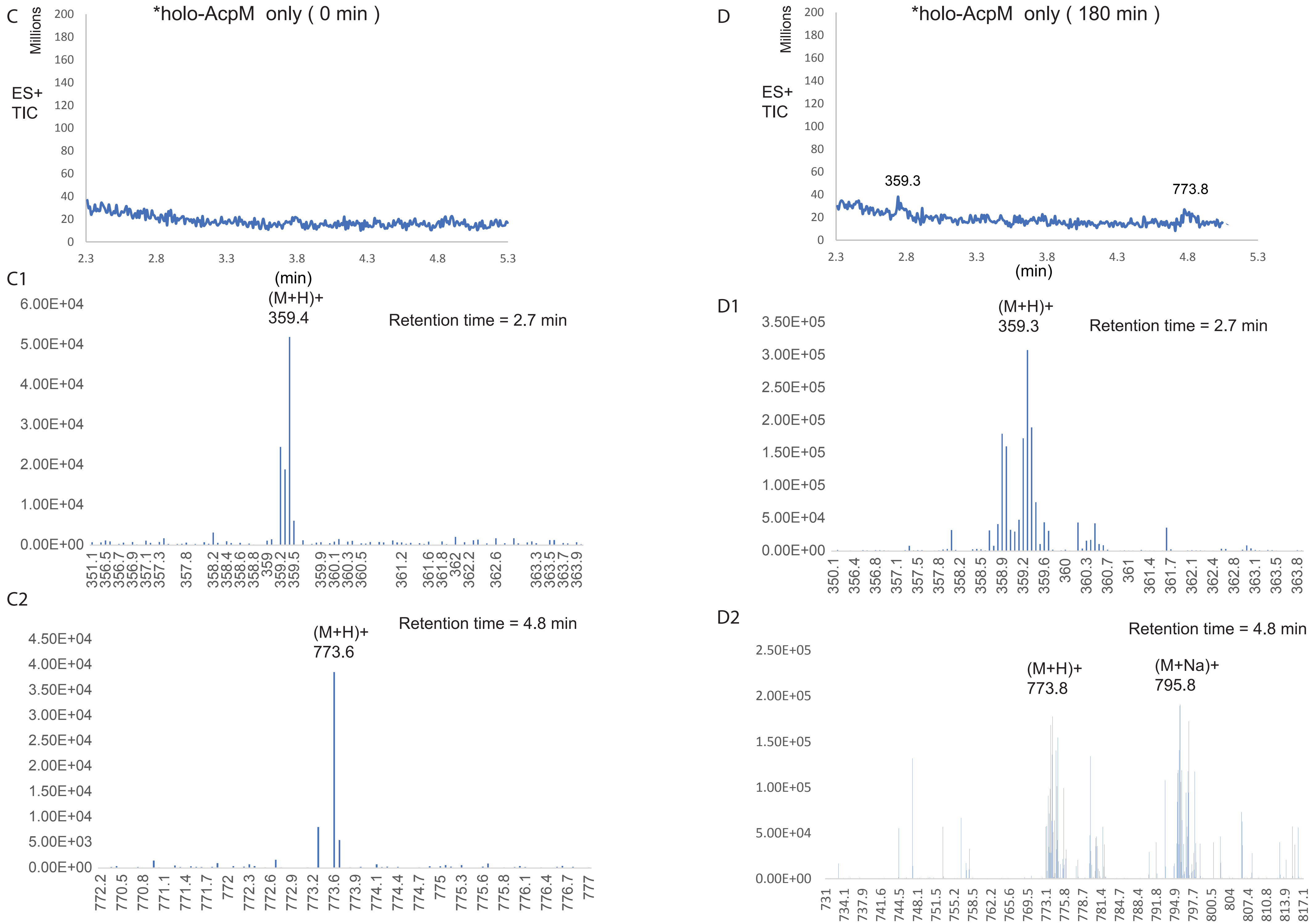

Figure S3. Mass spectrometry of PptH-catalyzed removal of *Ppt from *holo-AcpM. A-D) LCMS chromatograms charting the monitored enzymatic reactions using positive mode mass detection. The reactions were carried out for 0, 6, 30, 90 and 180 min. The lower chromatograms represent retention times of 2.7 and 4.8 min and correspond to the peaks with masses of 359 and 773 respectively. The superimposed structures (3B1, 3B2) depict both products that correspond to those masses. In addition to the $(M+H)^+$ ion, at times the $(M+Na)^+$ ion was detected as well. Experiments were performed two independent times.

Supplementary figure 4

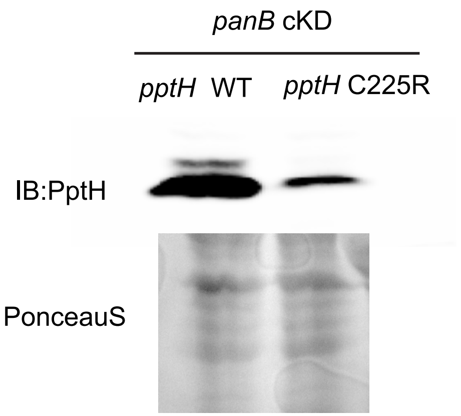

Figure S4. Level of PptH WT and PptH C225R in the *panB* cKD strain background. An equivalent amount of protein was loaded in each lane, also indicated by PonceauS staining (below).

Supplementary figure 5

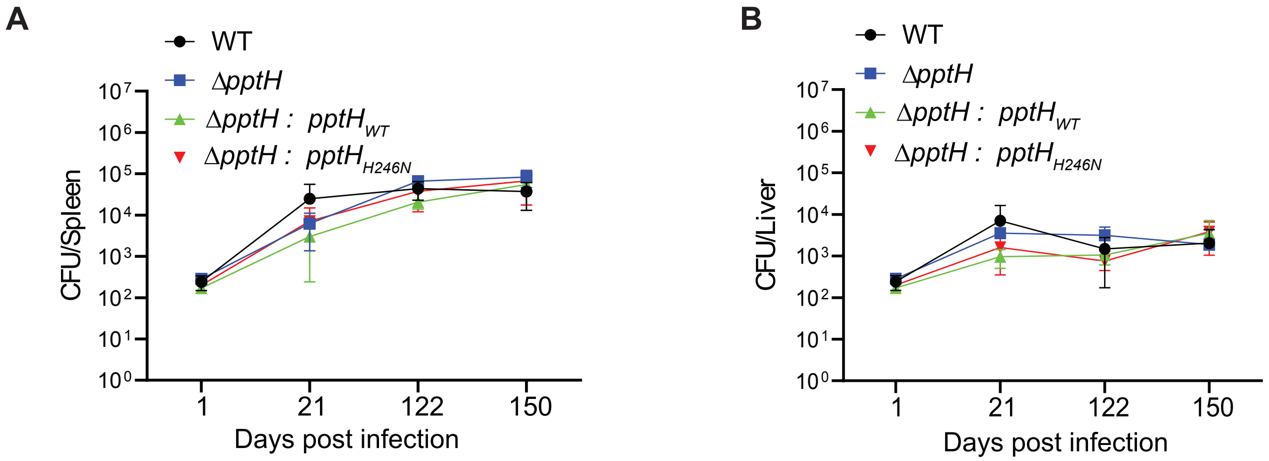

Figure S5. PptH is not required for growth and persistence in mice liver or spleen. A) Bacterial titers in spleen or B) in liver of C57BL/6 mice. Mice were infected with WT H37Rv, Δ*pptH*, Δ*pptH* : *ppt*H$_{\text{WT}}$ or Δ*pptH* : *pptH*$_{\text{H246N}}$ strains. Data are means ±SD from 5 mice per group per time point (4 mice at day 1) and are representative of two independent experiments.

Supplementary figure 6

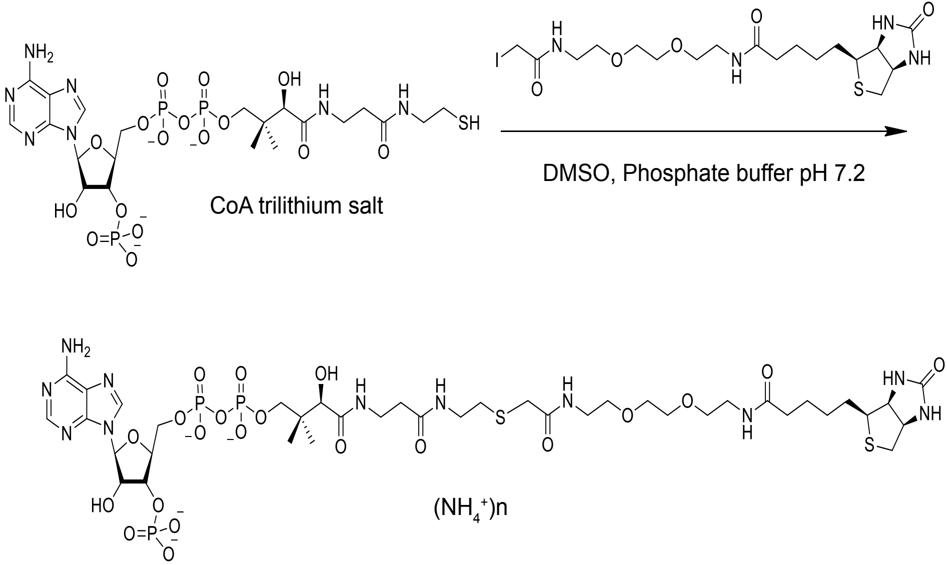

Biotin-PEG-ACM-CoA-Ammonium Salt
MW: 1182.05

Figure S6. Synthesis of Biotin-PEG-ACM-CoA Ammonium Salt

Table S1. Primers used for cloning of CPs

Targets	Forward primer	Reverse primer
*pks*13 *(M. tuberculosis)*	TACTTCCAATCCAATGCCATGGCTGACGTAGCG	TTATCCACTTCCAATGTTACTCGATGATCCGGGTG
entB (*E. coli*)	ATTGGATCCATGGCACCTATCCCCGCCAG	ATTAAGCTTTTACACCTCGCGGGAGAGTAGC
acpP (*E. coli*)	ATTGGATCCATGAGCACTATCGAAGAACGCG	ATTAAGCTTTTACGCCTGGTGGCCGTTGAT
acpP1 (*P. aeruginosa*)	ATTGGATCCATGAGCACCATCGAAGAACGC	ATTAAGCTTTTATTGCTGGTGAGCAACGAT
acpP (*K. pneumoniae*)	ATTGGATCCATGAGCACTATCGAAGAACGCG	ATTAAGCTT TTACGCCTGGTGGCCGTTGAT
